# What strategies are used to build practitioners’ capacity to implement community-based interventions and are they effective?: a systematic review

**DOI:** 10.1186/s13012-015-0272-7

**Published:** 2015-05-29

**Authors:** Jennifer Leeman, Larissa Calancie, Marieke A. Hartman, Cam T. Escoffery, Alison K. Herrmann, Lindsay E. Tague, Alexis A. Moore, Katherine M. Wilson, Michelle Schreiner, Carmen Samuel-Hodge

**Affiliations:** School of Nursing, University of North Carolina at Chapel Hill, Carrington Hall, CB #7460, Chapel Hill, NC 27599 USA; Center for Health Promotion and Disease Prevention, University of North Carolina at Chapel Hill, CB #7424, Chapel Hill, NC 27599 USA; Department of Nutrition, Gillings School of Global Public Health, University of North Carolina at Chapel Hill, 2200 McGavran-Greenberg Hall, CB #7461, Chapel Hill, NC 27599 USA; Department of Behavioral Sciences and Health Promotion, University of Texas School of Public Health, 7000 Fannin, Houston, TX 77054 USA; Department of Behavioral Sciences and Health Education, Rollins School of Public Health, Emory University, 1519 Clifton Road, NE, 5th Floor, Atlanta, GA 30322 USA; UCLA Kaiser Permanente Center for Health Equity, Fielding School of Public Health, University of California, Los Angeles, CA USA; Lineberger Comprehensive Cancer Center, University of North Carolina at Chapel Hill, CB# 7295, Chapel Hill, NC 27599 USA; Division of Epidemiology, Analysis and Library Services, Community Guide Branch, Centers for Disease Control and Prevention, 1600 Clifton Road NE, Mailstop E-69, Atlanta, GA 30333 USA

**Keywords:** Capacity building, Prevention support, Interactive Systems Framework, Technical assistance, Evidence-based practice

## Abstract

**Background:**

Numerous agencies are providing training, technical assistance, and other support to build community-based practitioners’ capacity to adopt and implement evidence-based prevention interventions. Yet, little is known about how best to design capacity-building interventions to optimize their effectiveness. Wandersman et al. (Am J Community Psychol.50:445–59, 2102) proposed the *Evidence*-*Based System of Innovation Support* (EBSIS) as a framework to guide research and thereby strengthen the evidence base for building practitioners’ capacity. The purpose of this review was to contribute to further development of the EBSIS by systematically reviewing empirical studies of capacity-building interventions to identify (1) the range of strategies used, (2) variations in the way they were structured, and (3) evidence for their effectiveness at increasing practitioners’ capacity to use evidence-based prevention interventions.

**Methods:**

PubMed, EMBASE, and CINAHL were searched for English-language articles reporting findings of empirical studies of capacity-building interventions that were published between January 2000 and January 2014 and were intended to increase use of evidence-based prevention interventions in non-clinical settings. To maximize review data, studies were not excluded a priori based on design or methodological quality. Using the EBSIS as a guide, two researchers independently extracted data from included studies. Vote counting and meta-summary methods were used to summarize findings.

**Results:**

The review included 42 publications reporting findings from 29 studies. In addition to confirming the strategies and structures described in the EBSIS, the review identified two new strategies and two variations in structure. Capacity-building interventions were found to be effective at increasing practitioners’ adoption (*n* = 10 of 12 studies) and implementation (*n* = 9 of 10 studies) of evidence-based interventions. Findings were mixed for interventions’ effects on practitioners’ capacity or intervention planning behaviors. Both the type and structure of capacity-building strategies may have influenced effectiveness. The review also identified contextual factors that may require variations in the ways capacity-building interventions are designed.

**Conclusions:**

Based on review findings, refinements are suggested to the EBSIS. The refined framework moves the field towards a more comprehensive and standardized approach to conceptualizing the types and structures of capacity-building strategies. This standardization will assist with synthesizing findings across studies and guide capacity-building practice and research.

**Electronic supplementary material:**

The online version of this article (doi:10.1186/s13012-015-0272-7) contains supplementary material, which is available to authorized users.

## Background

Public health and other community-based practitioners have access to a growing menu of evidence-based interventions (EBIs) to promote health and prevent disease. These EBIs include a range of programs, policies, and practices that have been shown to be effective at improving environments, behaviors, and health outcomes [1]. Practitioners continue to underuse prevention EBIs, in part because they lack the ability and motivation to do so [2, 3]. In response to this challenge, a growing number of agencies are intervening to build practitioners’ capacity [4, 5], which we define as the provision of ongoing support for the purpose of increasing practitioners’ awareness, knowledge, skills, self-efficacy, and motivation to adopt and implement EBIs [6]. Despite extensive investments in capacity building, little is known about how best to design capacity-building interventions to optimize their effectiveness [7].

Wandersman and colleagues have proposed two frameworks that describe constructs central to capacity building [8, 9]. The Interactive Systems Framework (ISF) for Dissemination and Implementation (2008) posits that transferring EBIs into practice requires interaction among three systems: (1) *prevention synthesis and translation systems* that disseminate EBIs (e.g., Guide to Community Preventive Services), (2) *prevention delivery systems* that use EBIs to promote health (e.g., community coalitions, health departments, community-based organizations), and (3) *prevention support systems* that bridge the gap between the two other systems by disseminating tools and providing training and technical assistance (TA) to build prevention delivery system capacity to effectively use EBIs in practice [8]. Prevention support systems build the organizational capacity of delivery systems and also build the capacity of public health practitioners, coalition members, and others working within those systems. Building on the ISF, Wandersman and colleagues (2012) proposed a second framework—the *Evidence*-*Based System for Innovation Support* (EBSIS). The EBSIS describes training, TA, tools, and quality assurance/quality improvement as four strategies that support systems use to build capacity, as well as salient variations in the way those strategies are structured according to their dosage, delivery mode, collaborative design, or proactive design [9].

Research has demonstrated that capacity-building interventions can be effective at increasing the adoption and implementation of EBIs [6, 7, 10], yet little is known about how best to design capacity building to maximize its effectiveness. The EBSIS offers a framework for building the evidence base to guide the design of capacity-building interventions. The purpose of this review was to contribute to the further development of the EBSIS by systematically reviewing empirical studies of capacity-building interventions to identify (1) the range of strategies used, (2) variations in the way they were structured, and (3) evidence for their effectiveness at increasing public health and other community-based practitioners’ capacity to adopt and implement evidence-based prevention interventions.

### Conceptual framework

**Fig. 1:**
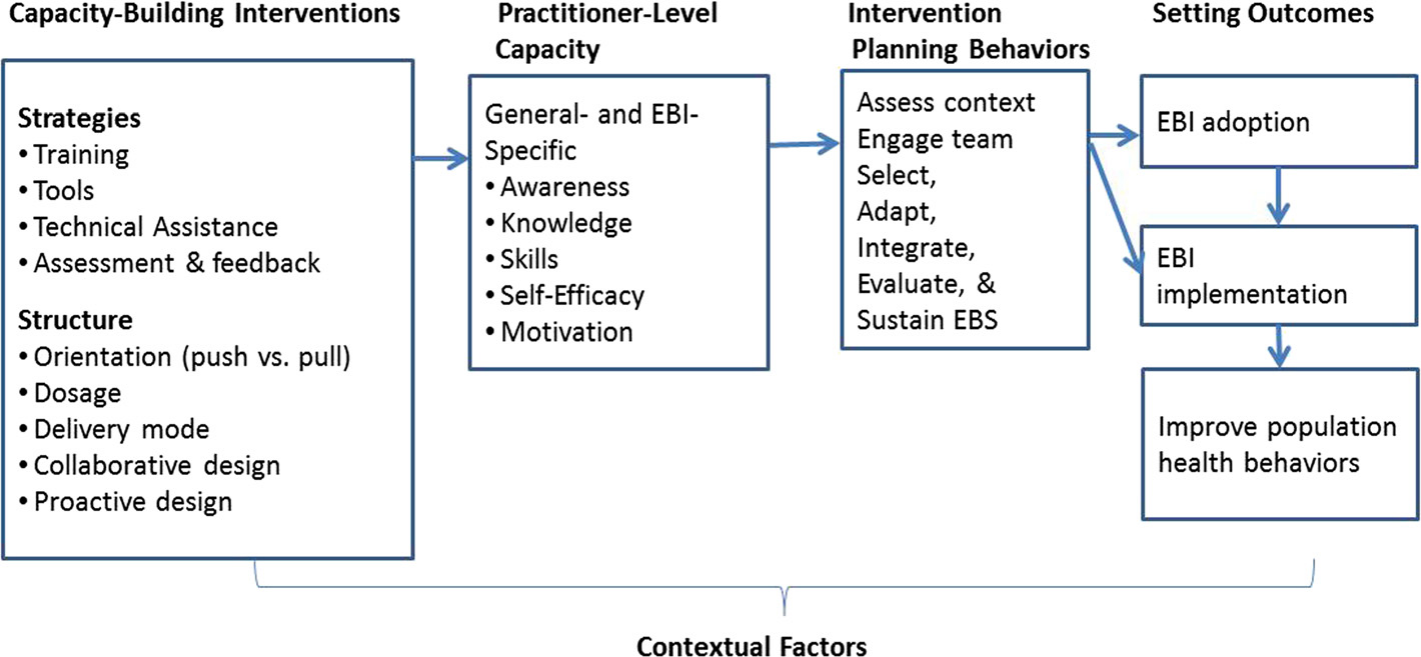
Conceptual framework of capacity-building interventions (adapted from the ISF and EBSIS) [8, 9]

We built on the EBSIS to create a conceptual framework to guide the review (Fig. 1). The framework includes capacity-building *strategies* and variations in the ways strategies are *structured* (see definitions of strategies and structures in Table 1.) The framework also describes intended *outcomes* (EBI adoption and implementation) and the mechanisms or mediating variables through which capacity building is hypothesized to affect those outcomes (practitioner capacity and EBI planning behaviors). *Practitioner capacity* is defined as the awareness, knowledge, skills, self-efficacy, and motivation to engage in EBI planning generally and/or to adopt and implement a specific EBI [6]. Although we recognize the importance of organization- and system-level capacity, the focus of the framework and this review is on practitioner-level capacity. *EBI planning behaviors* include collective behaviors such as assessing the community, identifying and prioritizing intervention options, developing an action plan, and evaluating processes and outcomes [6, 7, 10, 11].

**Table 1:** Definitions for key capacity-building constructs (adapted from EBSIS) [9]

Constructs	Definitions
Strategies	
Training	Pre-planned educational and/or skill-building sessions typically provided within group settings.
Tools	Defined by EBSIS as “informational resources designed to organize, summarize, and/or communicate knowledge.” We have revised this definition to include any electronic or print resource that practitioners might use to plan, implement, or evaluate an intervention.
Technical assistance	Interactive support that is individualized to the specific needs of individuals or teams. Those who provide TA may also be referred to as knowledge brokers, purveyors, linking agents, and external change agents among other terms.
Assessment and feedback	The support system’s strategy of monitoring and providing feedback on delivery system performance (used in place of the EBSIS term “quality improvement/quality assurance”)
Structures	
Orientation (push versus pull)	Support providers either build delivery system capacity to use a small number of pre-specified EBIs (push) or to use EBIs selected from a broad number of available EBIs (pull) [12].
Dosage	Comprises the duration, frequency, and amount of support provided with duration referring to the amount of time from the start to the end of support provision, frequency referring to how often support was provided during that time, and amount referring to the cumulative number of hours of support provided.
Delivery Mode	The communication channel used to deliver support (e.g., in-person, phone, Internet).
Collaborative design	The relationship between support providers and recipients varies in the extent of collaboration with some providers functioning as advisors while others function as fully engaged participatory partners.
Proactive design	TA is both anticipatory and responsive to recipients’ needs with TA providers initiating the process rather than simply reacting to TA requests.

*EBSIS* Evidence-Based System of Innovation Support [9], *TA* technical assistance, *EBIs* evidence-based interventions

The proposed conceptual framework modifies EBSIS terminology in three ways to fit the focus of the review. First, rather than the term “innovations”, we use the term “EBIs.” Both the ISF and EBSIS are used as frameworks for building capacity to use innovations, which are not restricted to EBIs and may include any practice that is new to practitioners. In contrast, the current review focuses on EBIs, which are defined as programs, policies, and practices that have been shown to be effective at improving environments, behaviors, and health outcomes [1].

Secondly, we use the term “assessment and feedback” to refer to the EBSIS strategy “quality assurance/quality improvement” because our terminology more clearly describes a strategy that might be employed in a capacity-building intervention as opposed to a strategy that might be employed by the delivery system to monitor and improve the quality of its own performance. Lastly, the framework also adds a distinction related to the overall “orientation (push versus pull)” of capacity building. Within a “push” orientation, support systems promote a small number of EBIs and build delivery systems’ capacity to use those EBIs. Within a “pull” orientation, the support system does not limit its focus to a few EBIs and instead builds delivery system capacity to select and use the EBIs that best fit their needs from a menu or from the universe of available EBIs [12]. Orientation (push versus pull) is added to the Framework (Fig. 1) as a variation in the structure of support.

We systematically reviewed tests of interventions to build practitioners’ capacity to adopt and implement community-based prevention interventions. The review addressed the following questions:

1. What types of capacity-building strategies are reported in the literature and how does their delivery structure vary across capacity-building interventions?
2. Are capacity-building interventions effective at improving practitioners’ capacity to use EBIs, their EBI planning behaviors, and their adoption and implementation of EBIs?
3. What contextual factors influence the design and effectiveness of capacity-building interventions?

## Methods

### Design

A systematic review of the literature was conducted by representatives of the Cancer Prevention and Control Research Network, a network of ten centers nationwide that is funded by the Centers for Disease Control and Prevention and the National Cancer Institute to accelerate the adoption and implementation of EBIs to prevent and control cancer, in partnership with a wide range of delivery systems [13]. Thus, members of the review team had extensive experience building practitioners’ capacity to use EBIs and were authors of a number of the publications included in the review.

### Search methods

**Fig. 2:**
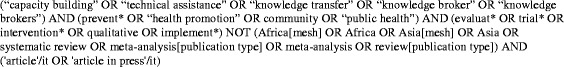
Full string of terms used to search PubMed

We searched PubMed, EMBASE, and CINAHL for peer-reviewed, English-language articles reporting the findings of studies of capacity-building interventions with a focus on interventions to promote public health and community-based practitioners’ use of prevention EBIs. The search was limited to articles published between January 2000 and January 2014 with the goal of assessing contemporary approaches to capacity building. We defined capacity building as the provision of interactive, ongoing support for the purpose of increasing practitioners’ ability and motivation to adopt and implement EBIs [6]. Community-based prevention EBIs were defined as EBIs that focused on primary prevention in a non-clinical context. In addition to the term “capacity building,” the search included terms commonly used to refer to ongoing interaction between support providers and recipients: “technical assistance” OR “knowledge transfer” OR “knowledge broker” OR “linking agent”. These terms were combined with the following: “community-based” OR “health promotion” OR “prevent*” OR “public health.” The search string also was designed to identify intervention studies and to exclude studies conducted in low and middle income countries because limitations in their financing, infrastructure, and information systems require distinct approaches to capacity building that fall outside the scope of this review [14]. The complete string of terms used to search PubMed is detailed in Fig. 2. Searching for literature in this area is difficult because the vocabulary has yet to be standardized [15]. To be more comprehensive, we also solicited recommendations from members of the Cancer Prevention and Control Research Network.

Two members of the research team reviewed the title and, as needed, the abstracts and full articles of identified publications. Articles were included if they were empirical studies of the provision of capacity-building interventions to promote the use of primary prevention EBIs in non-clinical settings. Capacity building had to be interactive and ongoing; thus, studies were excluded if they included only training or the online dissemination of information. We also excluded studies conducted in a single site, reports of “lessons learned” that lacked a description of the methods for collecting and analyzing data, and studies that only reported findings related to changes in population health behaviors/health status and did not include findings related to capacity, adoption, or implementation. To maximize the amount of data included in the review, we did not exclude studies a priori based on their design or quality. Instead, the following factors that contribute to validity were identified during abstraction: study design, sample size (practitioners and settings), data collection methods, and other factors (e.g., response rates). Potential threats to validity were taken into account in the report of synthesis findings [16].

### Data abstraction and synthesis

The findings from reports on capacity building are not amenable to meta-analysis due to their methodological and conceptual diversity. Therefore, quantitative (vote counting) [16] and qualitative (meta-summary) methods were used to summarize and synthesize both qualitative and quantitative findings [17]. Two reviewers independently abstracted the following information from each article: study characteristics (location, settings, participants, methods); focus of capacity building (EBIs, population behavior targeted); strategies and structure of capacity building (coded using Table 1 constructs); outcomes related to practitioners’ capacity (knowledge, skills, attitude, or beliefs) or intervention planning behaviors; outcomes related to setting/sector-level adoption and implementation of EBIs; and any qualitative findings related to variations in context and/or the structure of capacity-building strategies. To appraise potential threats to validity, the following data also were abstracted: design, sample size, and response rates [18]. The lead author trained all nine reviewers, who completed a pilot abstraction prior to participating in the review. The two reviewers for each publication compared their abstractions and resolved discrepancies by consensus.

Findings were then summarized and synthesized. The Table 1 codes were iteratively revised to capture the information derived from the review [19]. Vote counting methods were used to summarize data on the type and structure of capacity-building strategies and their effects on practitioner- and setting-level outcomes [16]. For findings on effectiveness, potential threats to validity were described as they related to each category of cumulative findings. Two authors applied meta-summary methods to iteratively review, summarize, and integrate qualitative findings into themes related to context and to variations in capacity-building strategies [17]. Once data were summarized and synthesized, findings were presented back to the full group of abstractors to ensure agreement.

## Results

**Fig. 3:**
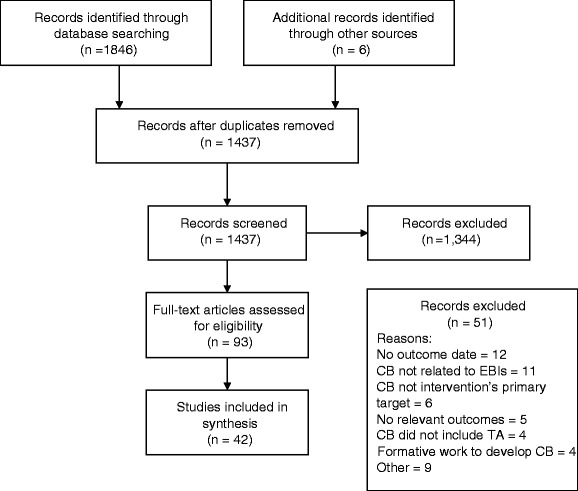
PRISMA flow diagram

The initial search yielded 1437 publications of which 42 publications reporting the findings from 29 studies met inclusion criteria (see Fig. 3 for PRISMA diagram). Table 2 provides an overview of the included studies. One study was conducted in Sweden and the remainder in the United States. A variety of frameworks and theories informed the studies included in the review. The three most frequently cited were the Diffusion of Innovations Theory (eight studies), the Interactive Systems Framework (seven studies), and Getting to Outcomes or Assets Getting to Outcomes (five studies). Others that were cited more than once included Social Cognitive Theory (three studies), Empowerment Evaluation (two studies), and Communities that Care (two studies).

**Table 2:** Description of publications included in review

Citation	Geographic location	EBIs	Population behavior targeted	Settings type and *n*	Practitioners type, *n*, and response rate (%)	Theories/frameworks	Capacity-building strategies
Group randomized trials							
Acosta et al. 2013; [43] Chinman et al. 2013 [37]	United States—ME	Unspecified EBIs	Youth problem behaviors	Community coalitions (*n* = 12), programs (*n* = 30)	Program directors (*n* = 32), coalition members (*n* = 303–376, 79–89 %)	Assets GTO, CFIR, Empowerment Evaluation Theory, SCT	Training, TA, tools
Buller et al. 2011 [49]	United States—CO, CA	Sun Safe Schools	Sun exposure	School districts (*n* = 112)	Superintendents, school board, school administrators (*n* = NS)	Diffusion of Innovations	TA, tools
Chinman et al. 2014 [38]	United States—SC	Beverage service training and compliance	Underage drinking	Coalitions (*n* = 6)	Coalition program directors (*n* = 6, 100 %)	ISF, GTO	Training, TA, tools
Crowley et al. 2012 [42]	United States—IA and PA	Unspecified EBIs	Youth substance abuse	Communities, public schools (*n* = 28)	School, substance abuse and mental health agency representatives (*n* = 422, 98 %)	ISF	Training, TA peer networking
Emmons et al. 2008 [25]	Unites States—MA	SunWise	Sun exposure	Schools (*n* = 28)	School nurses, health educators (*n* = 28, 76 %)	NS	Training, TA, tools
Escoffery et al. 2008, 2009; [60, 61] Glanz et al. 2005; [24] Hall et al. 2009; [62] Rabin et al. 2010 [33]	United States	Pool Cool	Sun exposure	Pools (*n* = 262–469 over 4 years)	Lifeguard, aquatic instructors (*n* = 43–2829, 54–80 %)	Diffusion of Innovations, Social Cognitive, and Organization Change Theories	Training, TA, tools
Fagan et al. 2012 [39]	United States—seven states	CTC prevention strategies	Youth substance use and other problem behaviors	Communities (*n* = 24)	School principals (153, 82 %), teachers (1664–1983, 75 %), program staff (326–393, 93 %)	CTC	Training, TA
Hannon et al. 2012 [26]	United States—WA	Community Guide EBIs	Healthy eating, physical activity, etc.	Mid-size employers (*n* = 48)	Human resources representatives (*n* = NS)	Diffusion of Innovations Theory, Social Marketing	TA, tools, peer networking
Kelly et al. 2000 [47]	United States—urban	SCT risk reduction model	HIV risk	AIDS Service Organizations (*n* = 74)	Aids service organizations directors and field staff (*n* = NS)	NS	Training, TA, tools
Little et al. 2013; [41] Rohrbach et al. 2010 [34]	United States	Project Toward No Drug Abuse	Substance abuse/violence	School districts (*n* = 10; 65 high schools)	Administrators (*n* = 41, 95 %), teachers (*n* = 50, 85 %)	ISF	Training, TA, tools, peer networking
Riggs et al. 2008; [30] Valente et al. 2007 [63]	United States—AR, CO, IA, MA, MO	Unspecified EBIs	Substance abuse	Cities/coalitions (*n* = 24)	Community leaders (*n* = 154–709; 36–95 %)	STAR, Diffusion of Innovations Theory	Training, TA, tools
Spoth et al. 2011 [36]	United Sates—IA, PA	Varied	Youth problem behaviors	Communities (*n* = 28)	Teachers, social service providers, other (*n* = 120, NS%)	PROSPER	Training, TA, tools
Group non-randomized trials							
Brownson et al. 2007 [44]	United States—nationwide	Community Guide EBIs	Physical activity	State/local health departments (eight states, overall *n* = NS)	State/local public health practitioners nationwide (*n* = 124–154, 73–94 %); course participants (*n* = 200)	Diffusion of Innovations Theory	Training, TA, tools
Chinmanet al. 2008; [20] Hunter et al. 2009a, [64] 2009b [65]	United States—CA and SC	Unspecified EBIs	Substance abuse	Community coalitions (*n* = 2; six programs)	Coalition participants (*n* = 268, 73–94 %), paid staff (*n* = 15–68), TA providers (*n* = 3)	GTO, ISF, Empowerment Evaluation Theory, SCT	Training, TA, tools
Elinder et al. 2012 [40]	Sweden	Unspecified EBIs	Obesity prevention	Schools (*n* = 18)	Health team members (*n* = NS)	Socio-ecological model, Local implementation logic model	Training, TA, tools, peer networking
Gingiss et al. 2006 [45]	United States—TX	Variety	Tobacco use	Schools (*n* = 134)	Principals (*n* = 109, 81 %) and health coordinators (*n* = 84, 63 %)	NS	Training, TA
Single group before-after study							
Batchelor et al. 2005 [46]	United States—TX	CDC’s compendium of effective interventions and others	HIV risk	Community planning groups (*n* = 6), HIV prevention agencies (*n* = 8)	Community planning group members (*n* = 25, 69 %), prevention providers (*n* = 112, 30 %)	Diffusion of Innovations	Training, TA, tools, peer networking
Beam et al. 2012, part 1 [22] and part 2 [23]	United States—nationwide	Unspecified EBIs	Obesity prevention	Schools (*n* = 1295)	School staff (*n* = NS in 1514 schools)	Healthy Schools Program, Diffusion of Innovations	Training, TA, tools consults with national experts
Brown et al. 2010, 2013; [27, 66] Feinberg et al. 2008 [35]	United States—PA	Unspecified EBIs	Youth problem behaviors	Community coalitions (*n* = 62–116)	Coalition board members, staff (*n* = 219–1624, 46–62 %)	CTC, Community Coalition Action Theory	Training, TA, tools, assessment and feedback
Duffy et al. 2012 [31]	United States—SC	Unspecified EBIs	Teen pregnancy prevention	CBOs (*n* = 11), Schools (*n* = 3)	Staff members of participating orgs (*n* = 13)	GTO, ISF	Training, TA, tools
Flaspohler et al. 2012 [28]	United States—OH and KY	Varied	Youth aggression, substance abuse	Elementary and middle schools (*n* = 12)	Core team at each school (a school administrator, class room teacher, one other rep) *n* = 5 schools, 15 people/year, NS%	GTO, ISF	Training, TA, tools, assessment and feedback assistance with data collection/analysis
Florin et al. 2012; [32] Nargiso et al. 2013 [51]	United States—RI	Unspecified environmental strategies	Substance abuse	Communities with high rates of alcohol and other drug use (*n* = 14)	Coalition designees (*n* = 14, 100 %), tobacco control coordinators (*n* = 9, 100 %)	Strategic Prevention Framework, ISF	Training, TA, tools, peer networking
McCracken et al. 2013 [67]	United States—SC	Three Cancer Control Planet EBIs	Diet, physical activity, cancer screening	CBOs (*n* = 3)	Lead CBO staff (*n* = 3, 100 %)	NS	TA
Philliber and Nolte 2008 [48]	United States—AZ, MA, SC, MN, NC	Unspecified EBIs	Teen pregnancy prevention	Coalitions (*n* = 8; three national, five state)	Program leaders (*n* = NS)	Diffusion of Innovations Theory	Training, TA, tools
Case study							
Cooper et al. 2013 [50]	United States—PA	Unspecified EBIs	Youth substance abuse and violence	CBOs (*n* = 77)	Mostly program directors (*n* = 77, 79 %)	NS	Training, TA
Harshbarger et al. 2006 [21]	United States	VOICES/VOCES	HIV risk	CBOs/Health Departments (*n* = NS)	CBO and health department staff (*n* = 162, 71 %)	NS	Training, TA, tools
Honeycutt et al. 2012 [68]	United States—GA	Treatwell 5-a-day, Body and Soul	Fruit and vegetable intake	Churches (*n* = 4), worksites (*n* = 3)	Six volunteers, two nurses, one RD, and one other (*n* = 10, NS%)	RE-AIM	TA, tools
Lee et al. 2011 [69]	United States—NC	A model curriculum	Tobacco use	Club houses for mentally ill (*n* = 9, 100 %)	Clubhouse staff and clients (*n* = 12, NS%)	NS	Training, TA, tools
Mihalic et al. 2008 [29]	United States	LifeSkills training	Substance use	Schools (*n* = 432, NS%)	Teachers (*n* = NS)	NS	Training, TA, tools

*CBO* community-based organization, *ISF* Interactive Systems Framework, *GTO* Getting to Outcomes, *SCT* Social Cognitive Theory, *CTC* Communities that Care

The most common settings for capacity-building interventions were communities (including those done with community-based coalitions; ten studies), schools (ten studies), and community-based organizations (five studies). Other settings included worksites, churches, pools, health departments, AIDs service organizations, and club houses for the mentally ill. The most frequently targeted behaviors included drinking and substance abuse (nine studies), sun exposure and youth problem behaviors (four studies each), HIV risk behaviors, healthy diet, physical activity, and tobacco use (three studies each). Study designs included 12 group randomized trials, 4 group non-randomized trials, 8 single group before-after studies, and 5 case studies that reported no pre-test or comparison group data.

Review findings are organized in response to the study’s research questions.

### What types of capacity-building strategies are reported in the literature?

The literature review confirmed that the EBSIS framework captured most of the strategies that were used to build practitioners’ capacity (see Additional file 1: Table S1). All 29 studies included *TA* as one of the capacity-building strategies. In the majority of studies, capacity building also included *training* (*n* = 27) and *tools* (*n* = 25). Among the tools described were manuals designed to guide practitioners in conducting an overall planning process (e.g., Chinman et al., [20]) or delivering a specific intervention (e.g., Harshbarger et al., [21]), e-newsletters (e.g., Beam et al., [22]; [23]), intervention materials (e.g., Glanz et al., [24]), evaluation tools (e.g., Emmons et al., [25]), and site-specific written recommendations (e.g., Hannon et al., [26]). Only three interventions included *assessment and feedback*; in each of these studies, capacity building included monitoring and feedback on the fidelity of EBI implementation [27–29].

The review identified two capacity-building strategies not described by the EBSIS: opportunities for peer networking and incentives. *Opportunities for peer networking* included bringing practitioners together to learn from each other via in-person trainings and TA sessions [28, 30–32], interactive conference calls [33], and web-based discussion forums [34]. Many of the capacity-building interventions provided *incentives* to motivate practitioners to participate in the capacity-building intervention or to adopt and implement EBIs, such as scholarships to participate in trainings [31], mandating participation in training as a requirement for funding [35], or provision of free resources (e.g., sunscreen to pool staff) [24].

### How does delivery structure vary across capacity-building interventions?

The way that capacity building was structured varied across the dimensions detailed in the EBSIS: orientation, dosage, delivery mode, and collaborative and proactive design (see Additional file 1: Table S1). In ten of the studies, the intervention *orientation* was towards “pushing” one or two specific EBIs (e.g., Pool Cool, VOICES/VOCES). In the remaining 19 studies, capacity building was oriented towards building practitioners’ capacity to “pull” EBIs from a wider range of options. *Dosage* varied widely, with authors often providing only limited information. In 19 of the studies, authors provided information on training dosage, typically in the format of number of trainings and their duration (e.g., 1 day). Authors provided almost no information on TA dosage in 15 studies. Authors did provide data on TA frequency (e.g., bi-weekly [29]) but not overall amount in three studies, overall amount of TA provided but not frequency (e.g., average of 76.2 h of TA per organization [31]) in five studies, and data on both the frequency and amount of TA provided in six studies. In some studies, authors provided overall exposure scores that combined dosage of training and TA with use of tools (e.g., Chinman et al., [20]). Authors also reported the *mode* through which TA was delivered, via face-to-face, by phone, or through combination of those media and email.

The *collaborative design* of capacity-building interventions also varied. The review revealed multiple related dimensions across which variation occurred in the collaborations between those providing and those receiving the capacity-building intervention. Those providing the intervention may work directly with delivery systems or may use a two-level or train-the-trainer model to build the capacity of intermediaries (e.g., field coordinators [24] or TA providers [36]) who then build the capacity of delivery systems. Capacity building also varied in whether it was provided to those who were planning or overseeing EBI implementation (e.g., members of a coalition [27]) versus those who actually delivered the EBI (e.g., teachers delivering a substance abuse intervention [34]). In most interventions, TA was provided *proactively*.

The review identified an additional dimension of variation in the structure of capacity building not specified by EBSIS. Capacity-building interventions varied in whether or not they were *delivered within the context of an overall planning model* (e.g., Getting to Outcomes). Planning models walk delivery systems through an overall planning process that includes stages such as assessing the context, selecting an EBI, implementing the EBI, and evaluating processes and outcomes. The most commonly used planning models were variations on Getting to Outcomes (*n* = 5 studies) [20, 28, 37, 38], Communities that Care (*n* = 2 studies) [27, 39], and one study each using PROSPER [36], the Healthy Schools Program [22], STAR [30], the Strategic Prevention Framework [32], and a locally developed logic model [40].

### Are capacity-building interventions effective at improving practitioners’ capacity to use EBIs, their EBI planning behaviors, and their adoption and implementation of EBIs?

As summarized in Table 3, studies were more likely to report findings related to effects on adoption (*n* = 12) or implementation (*n* = 11) than they were to report the effect that capacity building had on practitioners’ capacity (*n* = 7) or planning behaviors (*n* = 7) (see Additional file 2: Table S2 for a more detailed breakdown of findings for each study).

**Table 3:** Summary findings on effectiveness

	Number of studies	Number of significant versus not significant findings		
Category of finding		Significant within group difference	Significant between group difference	Significance not assessed
Effects on capacity	7^a^	2 of 2	2 of 6	1
Effects on EBI planning behaviors	7		2 of 3	4
Effects on adoption	12^a^	4 of 4	3 of 7	4
Effects on implementation	11^a^		5 of 6	6
Relationship between dose and outcomes	9	5 of 9		

*EBI* evidence-based intervention

^a^Studies report findings relevant to more than one column

### Effects on practitioners’ capacity to use EBIs

In the seven studies reporting findings related to intervention effects on capacity, findings were mixed. Studies reported response rates of 75% or better and had sample sizes of 120 or more, with the exception of Florin et al. (*n* = 9, [32]) and Little et al. (*n* = 50, [41]). Researchers operationalized capacity as self-efficacy (or self-report of skills), awareness, knowledge, attitudes, and/or beliefs about the value of the EBI. In two cases, group randomized trials found significantly greater improvements in capacity in the intervention versus the comparison group (self-efficacy [41] and knowledge [42]) and in two they did not (self-efficacy [43] and beliefs [41]). Neither of the two group non-randomized trials found significant between group differences in capacity (awareness [44] and self-efficacy and attitude [20]) following the intervention. Two studies found significant within group increases in capacity (awareness, skills [44] and self-efficacy [32]).

In one study, researchers analyzed the role that capacity played as both a mediator and moderator of capacity building’s effects on implementation [41]. They found that changes in practitioners’ self-efficacy (but not in their beliefs) mediated the effects that training and TA had on implementation fidelity. They also found that beliefs about an EBI’s value moderated the effects of Training/TA on EBI implementation fidelity, with Training/TA more effective for those practitioners who had more favorable beliefs at baseline.

### Effects on EBI planning behaviors

The review found mixed evidence for the effects that interventions had on practitioners’ collective EBI planning behaviors (see Table 3). Behaviors included, for example, developing an implementation plan and evaluating processes and outcomes [38]. Seven studies assessed planning behaviors. The unit of analysis for assessing behaviors was at the level of the setting (e.g., community, program, or school) and sample sizes ranged from 6 to 134. The three studies with the largest sample sizes tested for effectiveness. Of those, two group trials found significant differences, one randomized (*n* = 24, [30]) and one not (*n* = 134, schools [45]); and one group randomized trial found no significant differences in improvement between groups (*n* = 30 programs, [43]). In four studies, authors described improvements in planning behaviors without reporting significance [20, 31, 38, 46].

### Effects on adoption and implementation

The review found evidence that capacity building affects delivery system adoption and implementation of EBIs (Additional file 2: Table S2). Of the 12 studies that assessed *adoption*, four found a significant within group difference [22, 24, 26, 40] and two found significant between group differences in adoption rates with the intervention group having higher rates than the comparison [39, 47]; four additional studies found an increase in adoption following the capacity-building intervention without testing for significance [21, 25, 46, 48]. Findings were mixed, however, as four studies found non-significant group differences in adoption rates [24, 26, 44, 49]. Of the 11 studies that assessed effects on the extent or fidelity of *implementation*, all but one reported that capacity building had a positive effect. In five group trials (four of which were randomized), researchers compared differences between groups and found that the intervention group had better implementation outcomes than the comparison. In one of the five trials, findings were mixed and the intervention group performed significantly better on only some of the outcomes [39].

### Findings on the effects that variations in strategy type and structure have on outcomes

Review findings suggest that both the type and structure of prevention support strategies influence outcomes. In four studies, researchers compared the effectiveness of different combinations of prevention support strategies and found that interventions that provide TA in addition to training and tools have a greater impact on adoption and implementation [24, 33, 48, 50] than those that do not. In six of eight studies that assessed the relationship between dosage and outcomes, researchers found that dosage is related to the effect of prevention support on capacity [20, 43], planning behaviors [20, 43], adoption [22, 23], and implementation [32, 33, 50], such that higher dose or more engagement with the capacity-building intervention was associated with greater improvements in capacity, behaviors, adoption, and/or implementation (Additional file 2: Table S2). Riggs et al. [30] did not find a significant relationship between capacity-building dose and coalition capacity [30], and Spoth et al. [36] found no significant relationship between frequency of TA requests and the quality of planning behaviors or fidelity of implementation [36].

### What contextual factors influence the design and effectiveness of capacity-building interventions?

The review identified evidence on the following factors that may influence the types and structure of capacity-building strategies that will be most effective: setting capacity, attributes of the EBI, EBI fit with the setting, and the stage of the intervention planning process. In six studies, authors reported that *setting*-*level capacity* played an important role in determining the effects that capacity-building interventions have on adoption and implementation. Setting-level capacity included resources (e.g., time, staff, computers, funding, leadership) [20, 31, 41, 48] and collective attitude or willingness [28]. In prevention interventions, the “setting” may be the community, with a community coalition taking the lead in adopting and implementing EBIs as was the case in Brown et al. [27], which found that the quality of a coalition’s functioning (e.g., funding, leadership, internal and external relationships) was associated positively with the number of EBIs a coalition supported and their efforts to maintain implementation fidelity [27]. In a seventh study, Nargiso et al. [51] found that settings with lower initial capacity utilized more training and TA [51]. In two studies, authors suggested that *EBIs* with more components and less prescriptive implementation guidance are more difficult or require more effort to support than other EBIs [28, 32]. Study investigators also reported on the challenges created when EBIs did not *fit* a particular setting’s funding streams, values, or their clients’ cultures [26, 48]. Multiple studies reported differences in the types and/or amounts of prevention support required at different stages in the intervention *planning process* (e.g., Chinman et al. [20]).

## Discussion

**Fig. 4:**
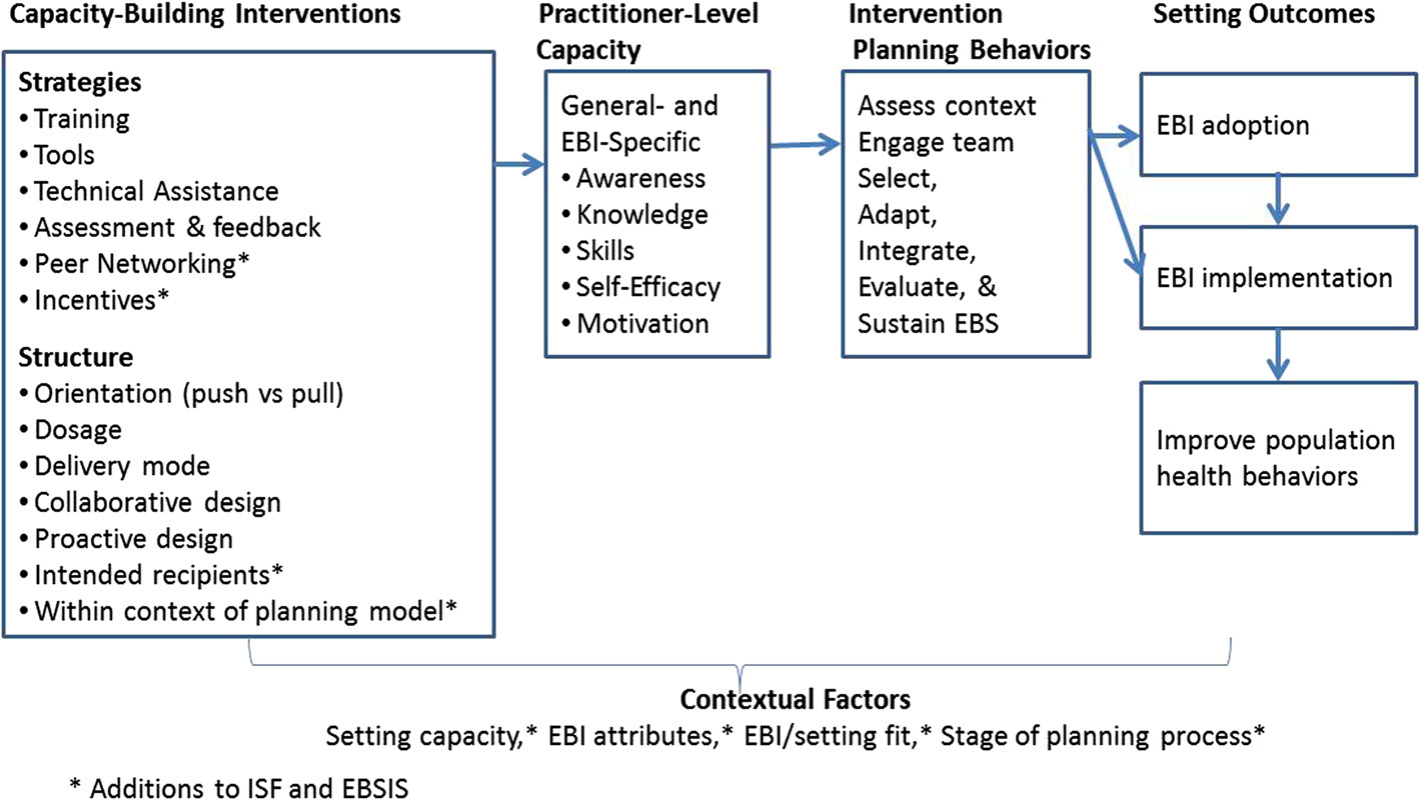
Revised framework for capacity-building interventions

Wandersman et al. [9] created the EBSIS as a framework to guide creation of an evidence base for capacity-building interventions [9]. To further advance the science of improving practitioners’ capacity, we have used an adapted EBSIS framework to guide a systematic review of the capacity-building literature. The findings from our review confirm the usefulness of EBSIS constructs and identify further refinements. Figure 4 presents the refined framework, which includes two new capacity-building strategies (peer networking and incentives) and two additional ways that the structure of those strategies differs across studies (intended recipients and whether provided within the context of a planning model). The review also provides details on the different types of tools being used to build practitioners’ capacity—a strategy that is only minimally described in the EBSIS framework. Lastly, the refined framework includes the review’s exploratory findings related to contextual factors that may moderate the effectiveness of capacity-building interventions and need to be considered in their design. These factors include setting capacity, attributes of the EBI, EBI/setting fit, and stage of the intervention planning process.

Similar to prior reviews, we found that capacity-building interventions can be effective at increasing EBI adoption and implementation [6, 7, 10]. These effectiveness findings are based on vote counting rather than meta-analysis and therefore should be interpreted with caution [52]. The review found that only seven of the 29 studies tested the effects that interventions had on capacity and only one assessed its role as mediator of an intervention’s effects on implementation. EBSIS posits that capacity is the primary mechanism through which prevention support affects the adoption and implementation of EBIs, and yet findings on interventions’ effects on capacity were mixed, with non-significant effects in three of the five studies that compared outcomes in the intervention group to a comparison group. Non-significant findings could have resulted from either limitations in the interventions or in the measurement of effects. Additional research focusing on identifying the capacities required to adopt and to implement EBIs and developing measures that are sensitive to change in those capacities will advance the science of capacity building. Limited evidence exists to support interventions’ effectiveness at improving practitioners’ intervention planning behaviors. Capacity was assessed at the level of individual practitioners, whereas planning behaviors were assessed at the organizational or coalition level, resulting in sample sizes that were often too small to assess for statistically significant change.

One of the review’s central purposes was to describe capacity-building strategies and synthesize evidence related to variations in their types and structures. The lack of information that authors provided about their capacity-building strategies and the way they were structured make it difficult to transfer successful strategies to new settings or to develop guidance for how best to structure capacity building. The need for specific guidance on how to structure capacity building is evidenced by findings that TA providers often experience their role as vague and ill-defined [53]. The lack of information related to capacity-building strategy types and structures also limits the potential to synthesize findings across studies. Use of standardized reporting such as CONSORT, TREND, or TIDieR may help practitioners or researchers disseminating results of interventions to include all relevant elements and ensure that reviewers can find more detailed information about intervention components [54, 55]. Synthesis is further constrained by the limited use of theory in the design and testing of capacity-building interventions.

### Limitations

The review of the literature was systematic but not comprehensive. Searches for literature related to implementation science are difficult because the field is still in the early stages of development, and consistent terminology has not been adopted [15, 56]. Because of these challenges, we cannot claim to have identified all reports of community-based interventions to build practitioners’ capacity to adopt and implement primary prevention EBIs. Of particular concern is the limited number of studies identified in countries other than the United States. The fact that a number of the reviewers were also authors on studies included in the review may have contributed to a biased conceptualization of what constitutes “capacity building.” However, having two individual abstract data from each article limited the potential for bias in data abstractions. The review’s findings were further limited by weaknesses in the included studies’ methods. Although 12 of the 29 studies were group randomized trials, small sample sizes limited the potential to identify significant differences between groups and the remaining studies employed weaker designs. In many of the publications, authors provided only limited detail on their interventions. Although this is similar to other types of intervention research where authors often include little detail on the dosage or mode of delivery [57], it limits analyses.

### Implications for future research

The EBSIS and our refined framework both move the field towards a more standardized approach to conceptualizing the types and structures of capacity-building strategies. This standardization will assist in synthesizing findings across studies and building the evidence base for what works under which circumstances. However, for findings to contribute to the evidence base, it is essential that researchers provide complete descriptions of how they designed and delivered their capacity-building interventions. Capacity building is a complex, behavioral change intervention. The prevention support field could benefit from guidance that the United Kingdom’s Medical Research Council and others have developed to facilitate the development, testing, and translation of complex interventions [58, 59]. Of greatest relevance to the present discussion are recommendations that researchers identify and evaluate the theory of change and provide detailed descriptions of the intervention “to enable replication, evidence synthesis, and wider implementation” (p. 2) [58].

EBSIS and the refined framework could also advance the field towards the development of theory. Since capacity is hypothesized as the primary causal mechanism, further research is needed to better understand the capacities that practitioners require to successfully adopt and implement EBIs and to develop measures of those capacities. Additional research is also needed to identify salient contextual factors that moderate the effects of prevention support and the best approaches to customizing prevention support contingent on those factors. The framework’s depiction of prevention support as a linear process is an oversimplification, and more research is needed to understand bi-directional interactions between support providers and practitioners and their effects.

## Conclusion

The number of researchers and agencies providing technical assistance and other capacity-building strategies to promote the use of evidence in practice is on the rise. However, the science to guide the design of prevention support is nascent. Only a limited number of researchers have taken a rigorous approach to designing, describing, and testing capacity-building strategies. As a result, little is known about how capacity-building strategies may vary across projects and how those variations may affect outcomes. This review contributes to the understanding of the types of capacity-building strategies and their effects thereby building the knowledge base on how to build practitioners’ capacity to use EBIs.

## Additional files

Additional file 1: Table S1. Content of support strategies used in publications included in this review. For each publication, details are provided on the planning model, training, technical assistance, tools, and other strategies used. Additional file 2: Table S2. Evidence for capacity-building intervention effectiveness. Evidence is summarized on each study’s findings on the effects capacity building had on capacity, planning behaviors, adoption, and implementation.

## Abbreviations

CBO: community-based organization
CONSORT: consolidated standards of reporting trials
CTC: Communities that Care
EBIs: evidence-based interventions
EBSIS: Evidence-Based System for Innovation Support
GTO: Getting to Outcomes
ISF: Interactive Systems Framework
SCT: Social Cognitive Theory
TA: technical assistance
TREND: transparent reporting of evaluations with non-randomized designs
