# Supplementary material for: What strategies are used to build practitioners’ capacity to implement community-based interventions and are they effective?: a systematic review
Source: Implement Sci. 2015 May 29;10:80. doi: 10.1186/s13012-015-0272-7 (PMC4449971; doi:10.1186/s13012-015-0272-7)
Supplement: Additional file 1: Table S1. — Content of support strategies used in publications included in this review. For each publication, details are provided on the planning model, training, technical assistance, tools, and other strategies used. [file 13012_2015_272_MOESM1_ESM.docx]

Online Supplement 1. Content of support strategies used in publications included in this review.

| **Citation** | **Planning Model** | **Training** | **TA** | **Tools** | **Other** |
| --- | --- | --- | --- | --- | --- |
| Acosta, 2013; Chinman and Acosta, 2013 | Assets Getting to Outcomes | In-person training (1 six-hour day) | Proactive, in-person, biweekly TA visits and as needed over 2 years; 511-612 hours overall (Mean=203 h, SD=237 h) | Manual and tools | Peer Networking |
| Batchelor, 2005 | No | 2-day training for planning group co-chairs; 5 session training for prevention providers | Ongoing TA (dose NS) | Assessment guide, EBI fact sheets, guidance on using assessment data |  |
| Beam, 2012, part 1 & part 2 | Healthy Schools Program change process | Train-the-trainer sessions (n=9), NS medium or duration | Proactive, in-person, phone calls, email exchange, dose varied | E-newsletters and online database containing more than 800 resources; 8 online topical tool kits | Consultations with national experts |
| Brownson, 2007 | No | In-person training, NS duration | TA (NS type, medium, dose) | CD Rom included CDC evaluation handbook, slide sets, grant related resources, selected websites |  |
| Brown, 2010, 2013; Feinberg, 2005, 2008, 2010 | Communities that Care | 3 multi-day trainings to coalitions. Coalitions then develop/coordinate trainings on EBIs for programs (NS medium, duration) | Proactive, In-person, phone, & email; in one study, on-site TA averaged 98-132 minutes monthly and distance TA averaged 70-73 minutes monthly. | Manual and assessment instruments | Incentives, fidelity monitoring of EBI implementation |
| Buller 2011 |  |  | email, in person, phone ongoing 2057 contacts (range 16-81 per district, mean 39; 19% interactive - most email | Binder of resources, website, meeting summary, e-newsletter |  |
| Chinman, 2008; Hunter 2009a, 2009b | Getting to Outcomes | Two one-day face-to-face trainings | Proactive, face-to-face, phone, and email. 1.5-2 hrs of TA weekly, followed by an additional 2 hrs of phone and email support. Total TA (hrs) = 78-322 per coalition (1-3 hours/week) | Manual, tools to assist with planning, implementation, evaluation, and sustaining |  |
| Chinman, 2014 | Getting to Outcomes | Classroom-based course (from 1 to 4 h) to retailors, Coalition training = 1-day (6 h) training | Proactive, face-to-face meeting about every other week.  612 hrs of TA (Mean=203 h, SD=237 h). | Manual, tools, and worksheets |  |
| Cooper, 2013 | No | Trainings, NS medium and duration | Yes, NS type, medium, dose |  | Incentives |
| Crowley, 2012 | No | Training, NS medium and duration | Proactive, at least weekly via bi-weekly phone calls, onsite consulting, email, learning community meetings |  | Learning community meetings |
| Duffy, 2012 | Getting to Outcomes | 1-day in-person training 2 times per year | Proactive, in-person, phone, and email, avg of 76.2h of TA per organization | Manual, tools, online database |  |

| Online Supplement 1. Content of support strategies used in publications included in this review (continued). | | | | | | |
| --- | --- | --- | --- | --- | --- | --- |
| Elinder, 2012 | Local logic model | | 4 workshops for health team members 4 training sessions for school staff | NS coaching | Needs assessment tool, planning guide, toolbox of written health education, other materials | Peer networking |
| Escoffery, 2008, 2009; Glanz, 2005; Hall, 2009; Rabin, 2010 | No | | 1 to 2 day in person training to field coordinators who provided in person training to pool staff (NS duration) | Researchers to field coordinators who provided to sites via email and 2-3 conference calls per summer, email primary medium with an average of 29.6/year between researchers and field coordinators, 5212 emails between field coordinators and sites over 4 years | Leader's guide for field coordinators; tool kit, Pool Cool curriculum, electronic copies of resources, physical materials (e.g., sunscreen), CD-ROM with resources for sites | Incentives to promote maintenance of Pool Cool |
| Emmons, 2008 | No | | Training | Three proactive 15-45 minute phone calls | Curricula, brochures, toolkit, evaluation tool to assess current school practices, menu of options |  |
| Fagan, 2012 | Communities that Care | | In-person training, NS duration | Annual proactive site visits and regular technical assistance via phone, email, and site visits |  | Incentives |
| Flashpohler, 2012 | Getting to Outcomes | | In person, quarterly | Core planning teams received "regular" on-site consultation |  | Community of practice, incentives, mandatory attendance of team at a request for application meeting, readiness assessment, assistance with data entry, cleaning, and analyses |
| Florin, 2012; Nargiso, 2013 | | Strategic Prevention Framework | In person trainings (duration not specified) | Group TA sessions. Mean amount Training/TA was 23.86 hours per task force | Workbooks and tools | Peer networking, tailored homework after in-person meetings |
| Gingiss, 2006 | | No |  |  |  | $2,000 annual reimbursement per school, contractual agreement required participation in trainings |
| Hannon, 2012 | |  |  | 3 in-person to Human Resource staff. Monthly proactive phone calls. Invited to contact as needed (few contacts). | Tailored recommendations, toolkits with ready to use materials |  |
| Harshbarger, 2006 | | No | 31 regional trainings for CBO and health department staff | TA available on request via phone or email | Implementation manual, videos, condom posters |  |

| Online Supplement 1. Content of support strategies used in publications included in this review (continued) | | | | | |
| --- | --- | --- | --- | --- | --- |
| Honeycutt, 2013 | No | Proactive TA - also responded to email and phone requests, | Bi-monthly proactive phone calls, 2 site visits, and as needed email and phone contacts. Total: 425 unique phone and e-mail contacts with grantees. (mean= 71 for churches and 37 for worksites) Over the 18 months of the project, staff spent a total of 47.4 hours providing TA, with each individual contact taking an average of 6.7 minutes. | NA | mini-grants |
| Kelly, 2000 | No | In-person, 2-day workshop | Monthly, proactive phone calls. Average length of calls = 26 minutes. | Manuals, physical materials, and instructional guides |  |
| Lee, 2011 | No | In-person, two-day staff and lead member training | Monthly, proactive in-person site visits and phone calls, email, and conference calls. During site visits, AHEC staff helped lead the group, modeling strategies for group facilitation. | Toolkit | Incentives |
| McCracken, 2013 | No |  | Yes, medium and dose NS |  |  |
| Mihalic, 2008 | No | 3-4 days of training | Annual site visits, as needed by phone, email or in-person, written feedback | Training and program materials |  |
| Philliber & Nolte, 2008 | No | Training to state and local organizations, dose and medium NS | Proactive, via regular phone calls, site visits, and in-person consultations | Communications materials | National & State conferences, Roundtables, and Networking and Information Sessions |
| Riggs, 2008; Valente, 2007 | STAR | Six interactive televised prevention trainings delivered to groups, with group discussion (2 hours each). | TA arm received proactive TA. Small amount of TA provided to training only and control conditions on request | Training Materials |  |
| Little, 2013; Rohrbach, 2010; | No | In-person, one-day training | Proactive for three sessions, then available on an "as-needed" basis thereafter | Online resources | Web-based discussion forum about implementation issues |
| Spoth, 2011 | PROSPER | NS | Proactive TA, dose NS | Intervention materials | Incentives |
