# Supplementary material for: What strategies are used to build practitioners’ capacity to implement community-based interventions and are they effective?: a systematic review
Source: Implement Sci. 2015 May 29;10:80. doi: 10.1186/s13012-015-0272-7 (PMC4449971; doi:10.1186/s13012-015-0272-7)
Supplement: Additional file 2: Table S2. — Evidence for capacity-building intervention effectiveness. Evidence is summarized on each study’s findings on the effects capacity building had on capacity, planning behaviors, adoption, and implementation. [file 13012_2015_272_MOESM2_ESM.docx]

**Online Supplement 2. Evidence for capacity-building intervention effectiveness**

| **Citation** | **Effects on capacity** | **Relationship between dose & outcomes** | **Effects on EBI planning behaviors** | **Effects on adoption** | **Effects on implementation** |
| --- | --- | --- | --- | --- | --- |
| **Group Randomized Trials** | | | | | |
| Acosta, 2013; [20] Chinman, Acosta, 2013 [21] | NS# self-efficacy | Being exposed vs. not exposed S related to self-efficacy & planning behaviors | NS# planning behaviors |  |  |
| Buller, 2011 [22] |  |  |  | NS# adoption of policies | S# strength of policies |
| Chinman, 2014 [23] |  |  | *Improved performance of planning behaviors |  |  |
| Crowley, 2012 [24] | S# knowledge |  |  |  |  |
| Emmons, 2008 [25] |  |  |  | *Higher adoption rates in schools that received TA versus those that did not |  |
| Escoffery, 2008, 2009; [26, 27] Glanz, 2005; [28] Hall, 2009; [29] Rabin, 2010 [30] |  | Dose NS related to implementation |  | NS#, Both groups S increased adoption of EBIs | S# implementation of Cool Pool components |
| Fagan, 2012 [31] |  |  |  | S# adoption of EBIs | S# reach, NS# implementation fidelity with 1 exception |
| Hannon, 2012 [32] |  |  |  | NS# adoption of EBIs. S increase in EBIs adopted. |  |
| Kelly, 2000 [33] |  |  |  | S# greater adoption rates in training & TA arm & training arm as compared to tools only |  |
| Little, 2013; [34] Rohrbach, 2010 [35] | S# self-efficacy, NS# beliefs |  |  |  | S# greater fidelity of implementation in Training & TA arm than in training alone |
| Riggs, 2008; [36] Valente, 2007 [37] |  | Training & TA dose NS related to general coalition capacities | S# Planning behaviors |  |  |
| Spoth, 2011 [38] |  | Frequency of TA requests NS related to planning behaviors or fidelity of implementation |  |  | *fidelity of EBI implementation and program reach |

* = significance not assessed, S = significant within group difference, S# = significant between group difference, NS = not significant**Online Supplement 2. Evidence for capacity-building intervention effectiveness (continued)**

| **Citation** | **Effects on capacity** | **Relationship between dose & outcomes** | **Effects on EBI planning behaviors** | **Effects on adoption** | **Effects on implementation** |
| --- | --- | --- | --- | --- | --- |
| **Group Non-Randomized Trials** | | | | | |
| Brownson, 2007 [39] | NS# awareness S knowledge, skills |  |  | NS# adoption of EBIs at state level S# increase in only one EBI at local |  |
| Chinman, 2008; [40] Hunter 2009a, [41] 2009b [42] | NS# self-efficacy, attitude | Dose S related to self-efficacy, attitude, & planning behaviors | *Programs in intervention improved more than those in comparison |  |  |
| Elinder, 2012 [43] |  |  |  | S adoption of health practices and environmental changes |  |
| Gingiss, 2006 [44] |  |  | S# Planning behaviors |  | S# extent of EBI implementation |
| **Single Group Before-After Study** | | | | | |
| Batchelor, 2005 [45] | *Knowledge, attitude |  | *Agency proposals inclusion of risk factor data | * EBIs adoption in intervention plans |  |
| Beam, 2012, part 1 [46] & part 2 [47] |  | Training dose S related to EBI adoption |  | S EBI adoption. |  |
| Brown, 2010, 2013; [48, 49] Feinberg, 2008 [50] |  | TA dose NS related to board functioning in subsequent year |  |  |  |
| Duffy, 2012 [51] |  |  | * Planning behaviors |  |  |
| Flaspohler, 2012 [52] |  |  |  |  | *extent of implementation |
| Florin, 2012; [53] Nargiso, 2013 [54] | S self-efficacy | Training & TA dose S related to policy enactment |  |  |  |
| McCracken, 2013 [55] |  |  |  |  | *program reach |
| Philliber & Nolte, 2008 [56] |  |  |  | *adoption of EBIs |  |

* = significance not assessed, S = significant within group difference, S# = significant between group difference, NS = not significant

**Online Supplement 2. Evidence for capacity-building intervention effectiveness**

| **Citation** | **Geographic location** | **EBIs** | **Population behavior targeted** | **Settings type, n, and response/ retention rate (%)** | **Practitioners type, n, and response rate (%)** |
| --- | --- | --- | --- | --- | --- |
| **Case Studies** | | | | | |
| Cooper, 2013 [57] |  | TA dose S related to sustaining EBIs |  |  |  |
| Harshbarger, 2006 [58] |  |  |  | *Sites adopting EBI |  |
| Honeycutt, 2012 [59] |  |  |  |  | *Fidelity of implementation of EBIs core components |
| Lee, 2011 [60] |  |  |  |  | *Fidelity of implementation of EBIs core components |
| Mihalic, 2008 [61] |  |  |  |  | *Fidelity of implementation |

* = significance not assessed, S = significant within group difference, S# = significant between group difference, NS = not significant.
